# A Role in Immunity for Arabidopsis Cysteine Protease RD21, the Ortholog of the Tomato Immune Protease C14

**DOI:** 10.1371/journal.pone.0029317

**Published:** 2012-01-06

**Authors:** Takayuki Shindo, Johana C. Misas-Villamil, Anja C. Hörger, Jing Song, Renier A. L. van der Hoorn

**Affiliations:** 1 The Plant Chemetics Laboratory, Chemical Genomics Centre of the Max Planck Society and Max Planck Institute for Plant Breeding Research, Cologne, Germany; 2 Center for Proteomics and Bioinformatics, Case Western Reserve University, Cleveland, Ohio, United States of America; University of Wisconsin-Milwaukee, United States of America

## Abstract

Secreted papain-like Cys proteases are important players in plant immunity. We previously reported that the C14 protease of tomato is targeted by cystatin-like EPIC proteins that are secreted by the oomycete pathogen *Phytophthora infestans* (*Pinf*) during infection. C14 has been under diversifying selection in wild potato species coevolving with *Pinf* and reduced *C14* levels result in enhanced susceptibility for *Pinf.* Here, we investigated the role C14-EPIC-like interactions in the natural pathosystem of Arabidopsis with the oomycete pathogen *Hyaloperonospora arabidopsidis* (*Hpa*). In contrast to the *Pinf*-solanaceae pathosystem, the C14 orthologous protease of Arabidopsis, RD21, does not evolve under diversifying selection in Arabidopsis, and *rd21* null mutants do not show phenotypes upon compatible and incompatible *Hpa* interactions, despite the evident lack of a major leaf protease. *Hpa* isolates express highly conserved EPIC-like proteins during infections, but it is unknown if these *Hpa*EPICs can inhibit RD21 and one of these *Hpa*EPICs even lacks the canonical cystatin motifs. The *rd21* mutants are unaffected in compatible and incompatible interactions with *Pseudomonas syringae* pv. *tomato*, but are significantly more susceptible for the necrotrophic fungal pathogen *Botrytis cinerea*, demonstrating that RD21 provides immunity to a necrotrophic pathogen.

## Introduction

Papain-like Cys proteases (PLCPs) are important players in plant immunity. PLCPs participate in immune responses and are targeted by pathogen-derived inhibitors. Cathepsin-B, for example, is a PLCP necessary to mount the full hypersensitive response (HR) in *Nicotiana benthamiana* [1]. Furthermore, RCR3 is a PLCP of tomato required to trigger HR in tomato plants carrying the *Cf-2* resistance gene, when infected by the fungal tomato pathogen *Cladosporium fulvum* producing Avr2 [2]. Similarly, PLCP RD19 is required in Arabidopsis for *RRS1-R*-mediated resistance against the bacterial pathogen *Ralstonia solanacearum* producing effector PopP2 [3].

A crucial role of PLCPs in disease immunity is also indicated by the observation that many pathogens produce effectors that manipulate these proteases. For instance, the PopP2 effector interacts with RD19 and causes the protease to relocalize from vesicles to the nucleus [3]. Furthermore, RCR3 is inhibited by Avr2, a small secreted protein produced by *C. fulvum* during infection of tomato [4]. Avr2 also inhibits PIP1, a closely related PLCP from tomato [5], [6]. Similarly, RCR3 and PIP1 are also inhibited by EPIC1 and EPIC2B, two closely related cystatin-like proteins, produced by the oomycete pathogen *Phytophthora infestans* during infection [7], [8].

We recently demonstrated that apart from RCR3 and PIP1, EPICs have an even higher affinity to the C14 proteases of tomato and potato [9]. This C14 protease has also been named TDI-65, CYP1 or SENU2 [10]–[13]. Silencing of a C14-like protease in *N. benthamiana* increases susceptibility for *P. infestans*, showing that this C14-like protease is indeed a *bona fide* target during infection [9]. Interestingly, these effector-targeted proteases have been under diversifying selection, indicating that they are part of an ongoing arms race at the plant-pathogen interface. Both RCR3 and PIP1, for example, carry a significant number of variant amino acids at the surface where Avr2 or EPICs are presumed to interact, and at least one of these variant residues can suppress inhibition by Avr2 [5]. Furthermore, C14 exhibits a pattern of diversifying selection in wild potato, which are the natural hosts of *P. infestans* [9], illustrating that diversifying selection correlates with coevolving plant-pathogen interactions. Interestingly, some of the variant residues in potato C14 seem to locate at the predicted EPIC interaction surface in 3D modelling studies [14]. Similar traces of molecular arms races have been described for other enzyme-inhibitor interactions at the plant-pathogen interface [15].

In this study, we studied the role of the C14-like protease in *Arabidopsis thaliana* and investigated if an interaction orthologous to EPIC-C14 would exist in the model pathosystem *Hyaloperonospora arabidopsidis* (*Hpa*) and *A. thaliana*. *Hpa* is an obligate biotrophic oomycete pathogen that causes downy mildew on Arabidopsis [16]. As a natural pathogen, *Hpa* has co-evolved with Arabidopsis and exists as ecotype-specific isolates [17]. The genome sequence of *Hpa* revealed the loss of genes that encode hydrolytic enzymes or proteins triggering host cell death, consistent with the lifestyle of *Hpa* as a stealthy pathogen that does not kill host cells [18]. During infection, *Hpa* grows in the leaf apoplast and employs haustoria to feed from parenchyma cells [16]. Since *C. fulvum* and *P. infestans* both colonize the host apoplast and encounter similar apoplastic Cys proteases, we investigated the role of EPIC-C14-like interactions in the *Hpa*-Arabidopsis pathosystem. In addition, we tested the role of the C14-like protease in other pathogens of Arabidopsis.

## Results

### Arabidopsis RD21 is the ortholog of tomato C14

**Figure 1:**
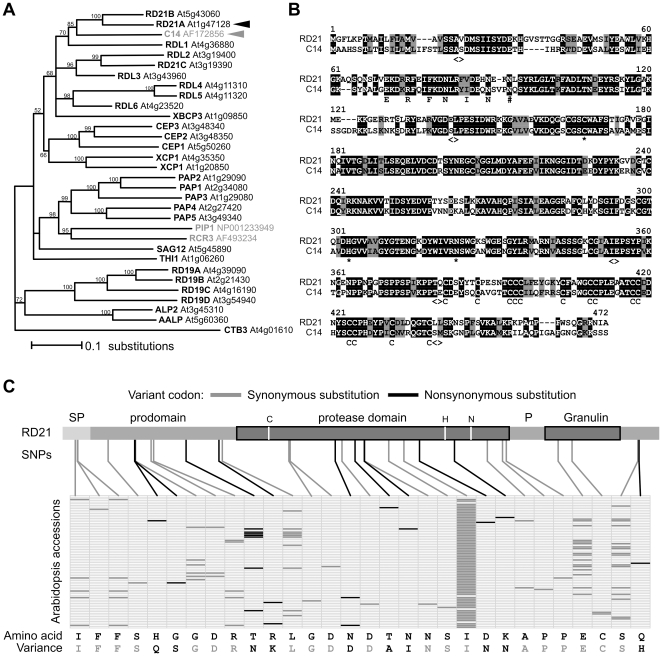
Arabidopsis RD21 is the ortholog of tomato C14. **A,** Phylogenetic tree of protein sequences of Arabidopsis papain-like Cys proteases (black) and tomato C14, RCR3 and PIP1 (grey). This tree was obtained using the neighbor joining method with bootstrap support. Arabidopsis CTB3 was used as outgroup and CTB1 and CTB2 were not included in the analysis. **B,** Alignment of Arabidopsis RD21 with tomato C14 protein sequences. *, catalytic residues; #, putative glycosylation site; C, cysteine; <>, transition between protein domains. **C,** Natural variation of *RD21A* in 80 Arabidopsis accessions. The position of the single nucleotide polymorphisms (SNPs) in the RD21 open reading frame are indicated on top, and their distribution in the ecotype sequences are indicated in the matrix. Nonsynonymous SNPs are indicated in black and synonymous SNPs in grey, respectively. The affected amino acids are summarized on the bottom. See Figure S2 for more details.

To identify the ortholog of tomato C14 in the Arabidopsis genome, we performed a BLASTP search of the C14 protein sequence to the Arabidopsis protein TAIR10 database and found that RD21 (encoded by *RD21A* gene, At1g47128) is the closest ortholog of tomato C14 (E-value e-162). Phylogenetic analysis of the pro- and protease domains of Arabidopsis PLCPs demonstrate that not only the presence of the granulin domain, but also the proprotease of RD21 is most closely related to tomato C14 (**Figure 1A**). The protein alignment shows that RD21 and C14 are 58%, 76% and 61% identical in the prodomain, protease domain and granulin domain, respectively (**Figure 1B**). Similar to tomato *C14*, Arabidopsis *RD21A* is highly expressed in leaf tissue and is upregulated during senescence [19]. The closest homolog of RD21 in Arabidopsis is RD21B (At5g43060), which also carries a granulin domain but the corresponding gene is transcriptionally less expressed in leaves when compared to *RD21A* (**Figure S1**) and has not been detected in leaf extracts by protease activity profiling [6], [20]. We therefore focussed our studies on RD21 (At1g47128) as the ortholog of tomato C14.

To evaluate whether selective forces are acting on the *RD21A* gene similar to potato *C14*, we performed population genetic analyses with 80 *RD21A* alleles extracted from genome sequences of 80 *Arabidopsis thaliana* accessions [21]. When combined, the 80 *RD21A* sequences contain 30 single nucleotide polymorphisms (SNPs), which affect 27 codons in the 1389 bp long open reading frame of *RD21A* (**Figure 1C** and **Figure S2**). Only ten SNPs affect the encoded amino acid but most of these variant residues are similar (**Figure 1C** and **Figure S2**). The nonsynonymous nucleotide diversity (π_a_ = 0.00041) is lower when compared to the synonymous nucleotide diversity (π_s_ = 0.0054) and the π_a_/π_s_ ratio is 0.077, which indicates that *RD21A* is under purifying selection.

We also calculated Tajima's *D* (*D*_T_) and Fu and Li's *D* (*D*_F_), which summarize the site frequency spectrum of SNPs and can indicate selective pressures or demographic effects acting on *RD21A*. Both values are significantly negative compared to the expectation under neutrality (*D*_T_ = −1.912, *P*<0.05 and *D*_F_ = −3.22, *P*<0.02). This negative deviation from neutrality is caused by an excess of SNPs that occur with low frequencies (**Figure 1C** and **Figure S3**). In fact, the majority (seven of the ten) of the nonsynonymous mutations occur in only one *RD21A* sequence (**Figure 1C** and **Figure S3**). Even though these data do not exclude that the observed pattern is caused by demography, these findings indicate that natural variation at the *RD21A* gene is not maintained by balancing selection.

To extend the analysis of SNPs we also included the RD21-encoding sequence of *Arabidopsis lyrata* (gi|297852301). *A. lyrata RD21A* differs at 54 SNPs from *A. thaliana RD21A* Col-0, which affect 52 codons (**Figure S4**). Importantly, 39 of these altered codons encode an invariant amino acid, and only 5 codons affect the amino acid code significantly, of which four are located in the signal peptide and prodomain (**Figure S4**). The ratio of nonsynonymous to synonymous divergence K_a_/K_s_ of this comparison is 0.087. This result confirms the conservation of RD21, even in comparisons between species.

### EPIC orthologs are present in *Hpa* isolate Emoy2

**Figure 2:**
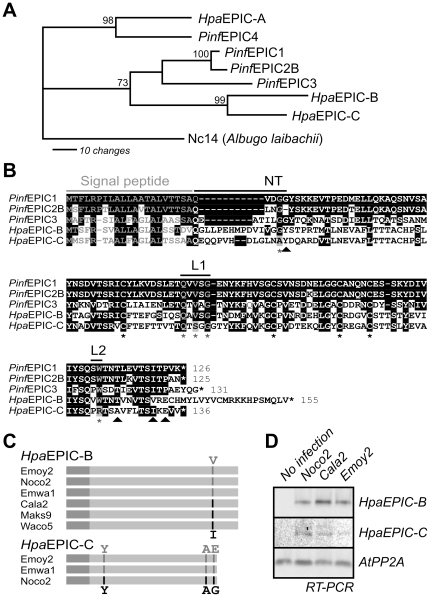
EPIC-like proteins from *Hpa*. **A,** Phylogenetic relationship between EPIC proteins. One of 1,000 most parsimonious trees showing the relationship between *Pinf* and *Hpa* EPICs. This tree was obtained by heuristic search with bootstrap support. A cystatin from *Albugo laibachii* was used as outgroup. **B,** Protein sequence alignment of *Hpa*EPIC-B and -C with *Pinf*EPIC1, -2B and 3. *, functionally important residues; NT, N-terminus; L1, loop-1; L2, loop-2. Triangles indicate amino acids at variant codons. **C,** Distribution of four variant codons found in *HpaEPIC-B* and *-C* sequences of various *Hpa* isolates. The variant codons are indicated with grey and black lines and the amino acid encoding the codons are indicated on the top and bottom. **D,** *HpaEPIC-B* and *-C* are expressed during infection. RNA was isolated from Arabidopsis plants infected with *Hpa* isolates Noco2, Cala2 or Emoy2 at 5 dpi and used as template for RT-PCR with specific primers for *HpaEPIC-B* and *-C*.

To identify EPIC orthologs from *Hpa*, we performed a BLASTP search with *Pinf*EPICs on the genome database of *Hpa* isolate Emoy2 at the VBI Microbial Database [18] and identified three EPIC-like proteins, called *Hpa*EPIC-A, -B and -C. Phylogenetic analysis of the *Pinf* and *Hpa* cystatins showed that *Hpa*EPIC-A is most closely related to *Pinf* EPIC4, and that *Hpa*EPIC-B and -C are more similar to *Pinf* EPIC1, -2B and -3 (**Figure 2A**). Thus, *Hpa*EPIC-B and -C are the most likely orthologs of *Pinf*EPIC1 and -2B, and were selected for further studies.

Alignment of *Hpa*EPIC-B and -C with *Pinf*EPIC1, -2B and -3 shows that *Hpa*EPIC-B contains the classical conserved cystatin features: a conserved glycine in the N-terminus; a conserved QxVxG motif in the middle, and a conserved tryptophane in the C-terminus (**Figure 2B**). Thus, *Hpa*EPIC-B is likely to have cystatin activity. In contrast, *Hpa*EPIC-C carries a QxSxG instead of the QxVxG motif, and lacks the conserved glycine and tryptophane in the N- and C-termini, respectively (**Figure 2B**). The striking disruption of the three key cystatin motifs is likely to render *Hpa*EPIC-C inactive as a Cys protease inhibitor.

### *Hpa*EPICs are conserved and expressed in other *Hpa* isolates

To investigate if *HpaEPIC-B* and *-C* are also present in other *Hpa* isolates and to determine natural variation in these genes, *HpaEPIC-B* and *-C* were amplified and sequenced from various isolates using PCR on genomic DNA, isolated from infected plants. Isolates Noco2 and Emwa1 contain an *HpaEPIC-B* that is identical to that of the Emoy2 isolate, whereas the *HpaEPIC-B* of isolates Cala2, Maks9 and Waco5 carry a single nucleotide polymorphism resulting in a V133I substitution (**Figure 2C** and **Figure S5**). *HpaEPIC-C* of Emwa1 is identical to that of Emoy2, and differs at three sites from that of the Noco2 isolate, causing two silent mutations and one amino acid substitution (E134G), located in the C-terminus of *HpaEPIC-C* (**Figure 2C** and **Figure S6**). Importantly, the disrupted cystatin motifs in *Hpa*EPIC-C are conserved among the *Hpa* isolates. The low diversity in *HpaEPICs* suggests strong conservation of these proteins in the investigated *Hpa* isolates.

To detect the expression of *HpaEPICs* during infection, RNA was isolated from Arabidopsis plants infected with isolates Emwa2, Noco2 and Cala2, and used as template for RT-PCR using *HpaEPIC*-specific primers. Both *HpaEPIC-B* and *HpaEPICB-C* were detected in the infected material and not in non-infected plants (**Figure 2D**), indicating that both genes are expressed during infection. Poor amplification of *Hpa*EPIC-C suggests that transcript levels of this gene are relatively low when compared to that of *Hpa*EPIC-B.

### Characterization of Arabidopsis *rd21* mutant lines

**Figure 3:**
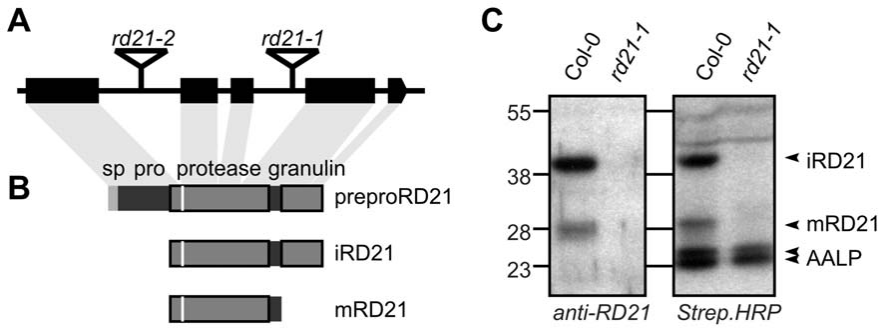
RD21 structure and knock-out lines. **A,** Gene structure of *RD21A* (At1g47128). The *RD21A* gene consists of 5 exons. Mutants *rd21-1* (SALK_90550) and *rd21-2* (SALK_65256) contain T-DNA insertions in the third and first introns, respectively. **B,** Domains encoded by the *RD21A* open reading frame. The RD21 protein consists of a signal peptide (sp, left), an autoinhibitory prodomain (pro), a protease domain with catalytic cysteine (white stripe), and a granulin domain (right). RD21 exists in two active isoforms: the granulin-containing intermediate (i) RD21, and the mature (m) RD21 lacking the granulin domain. **C,** The *rd21-1* line is a null mutant. The *rd21-1* mutant lacks iRD21 and mRD21 proteins (left) and the major upper signals in the protease activity profile (right). Leaf extracts of Col-0 and *rd21-1* mutant plants were labelled with DCG-04 and proteins were detected with RD21 antibody and streptavidin-HRP. A remaining signal at 30 kDa is sometimes visible in the *rd21* mutant lines (**Figure S6**), and can contain CTB3, XCP2 and XCP1 [20].

To investigate the role of *RD21A* in the Arabidopsis-*Hpa* interaction, we characterized the *rd21-1* knockout line [22], and selected a second, independent knockout line, *rd21-2*. The *rd21-1* and *rd21-2* lines carry T-DNA insertions in the third and first introns of *RD21A*, respectively (**Figure 3A**). The *RD21A* gene encodes a pre-pro-protease carrying a C-terminal granulin domain (**Figure 3B**). RD21 matures in several steps, resulting in a 40 kDa intermediate RD21 (iRD21) and 30 kDa mature RD21 (mRD21) [23]. Both iRD21 and mRD21 are active proteases and only iRD21 carries the C-terminal granulin domain. Western blot analysis of leaf extracts from these plants shows signals at 30 and 40 kDa, representing mRD21 and iRD21, respectively (**Figure 3C**; [23]). Both 30 and 40 kDa RD21 signals are absent in the *rd21-1* and *rd21-2* mutants (**Figure 3C** and **Figure S7**), demonstrating that these mutants are null mutants. Protease activity profiling using the biotinylated DCG-04 probe [20], [24] shows that the 40 kDa signal and the upper 30 kDa signals in the activity profile are absent in the *rd21* mutants (**Figure 3C** and **Figure S7**), indicating that RD21 is one of the major Cys protease in leaf extracts and that no compensatory Cys protease activities are detectable in these knock-out lines. Importantly, despite severe defects in Cys protease activities, no macroscopic phenotypes were observed for the *rd21* mutants grown in the greenhouse.

### Lack of RD21 does not affect *Hpa* infections

**Figure 4:**
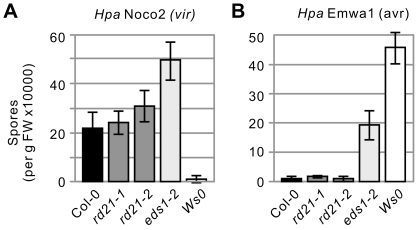
Mutant *rd21* lines are unaltered in their interactions with *Hpa*. **A,** Compatible *Hpa* interactions. (Mutant) Arabidopsis seedlings were infected with *Hpa* isolate Noco2, which is virulent on Col-0 but not on Ws. Spores were counted at 7 dpi in triplicate. Error bars represent standard deviation (SD) of three independent spore isolations. This experiment is repeated once with similar results. **B,** Incompatible *Hpa* interactions. (Mutant) Arabidopsis seedlings were infected with *Hpa* isolate Emwa1, which is virulent on Ws-0 but not on Col-0. Spores were counted at 7 dpi in triplicate. Error bars represent SD of three independent spore isolations.

To investigate the role of RD21 in the *Hpa*-Arabidopsis interactions, (mutant) Arabidopsis plants were infected with *Hpa* isolate Noco2, which is virulent on Arabidopsis ecotype Col-0. Unexpectedly, infection on *rd21* lines occurs indistinguishable from that of the Col-0 control and spore counts at 7 days-post-inoculation (dpi) repeatedly revealed no significant differences of infection of *rd21* mutant lines when compared to the Col-0 control (**Figure 4A**). In contrast, *eds1-2* mutant facilitates significant growth of *Hpa*Noco2, as reported previously (**Figure 4A**).

Since RD21 is an abundant vacuolar protease [23], [25], and the vacuolar content is released into the apoplast during the hypersensitive response (HR) [26], we anticipated that RD21 might be acting during incompatible interactions. We therefore infected the (mutant) plants with *Hpa* isolate Emwa1, which triggers *RPP4*-dependent HR on Col-0 [17]. Spore counts at 7 dpi showed no significant differences between *rd21* mutant plants and *Col-0* wild-type plants, whereas the controls (*eds1-2* and ecotype *Ws*), are susceptible (**Figure 4B**). Taken together, we observed no significant phenotype for *rd21* mutants upon infection with virulent and avirulent *Hpa* isolates.

### Mutant *rd21* plants show no phenotype in *P. syringae* infections

**Figure 5:**
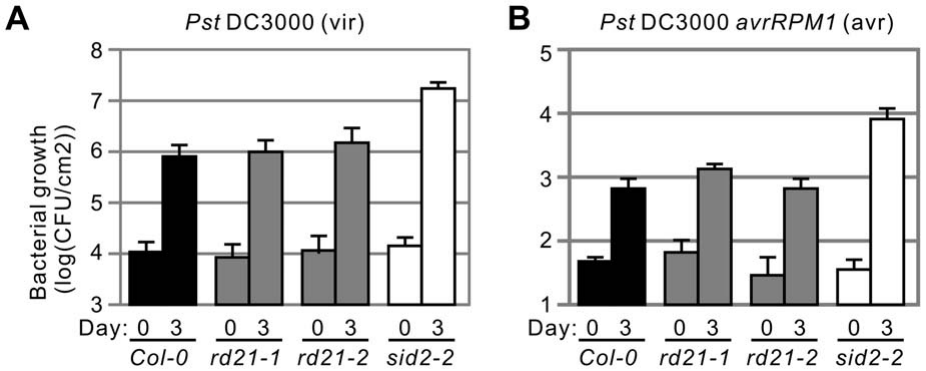
Mutant *rd21* lines are not compromised in interactions with *Pst*DC3000. **A,** Compatible *Pst* interactions. (Mutant) Arabidopsis plants were spray-inoculated with *Pst*DC3000 (vir) and bacterial populations were measured at 0 and 3 dpi. Error bars represent SD of 5 independent bacterial extractions. This experiment was repeated three times with similar results. **B,** Incompatible *Pst* interactions. (Mutant) Arabidopsis plants were spray-inoculated with *Pst*DC3000 avrRpm1 (avr) and bacterial populations were measured at 0 and 3 dpi. Error bars represent SD of 5 independent bacterial extractions. This experiment was repeated three times with similar results.

Having the *rd21* mutant lines at hand, we decided to test if the plants show any phenotype with other pathogens. We first tested infection by the virulent *Pseudomonas syringae* pv. *tomato* DC3000 (*Pto*DC3000). The *sid2-2* mutant line was included in this assay as positive control since this line supports enhanced bacterial growth due to its incapability to accumulate inducible salicylic acid (SA) [27]. The *rd21* mutant supports *Pto*DC3000 growth undistinguishable from wild-type plants, whereas the *sid2-2* mutant is more susceptible (**Figure 5A**).

To test if RD21 plays a role during infection with avirulent bacteria, we challenged the (mutant) plants with *Pto*DC3000 expressing *avrRpm1*. Again, bacterial populations were indistinguishable between *rd21* mutant and wild-type lines, whereas bacterial growth was enhanced in the *sid2-2* mutant (**Figure 5B**). In conclusion, no bacterial growth phenotypes were observed for *rd21* mutant lines upon infection with virulent or avirulent *Pto*DC3000 bacteria.

### Lack of RD21 increases susceptibility for *Botrytis cinerea*

**Figure 6:**
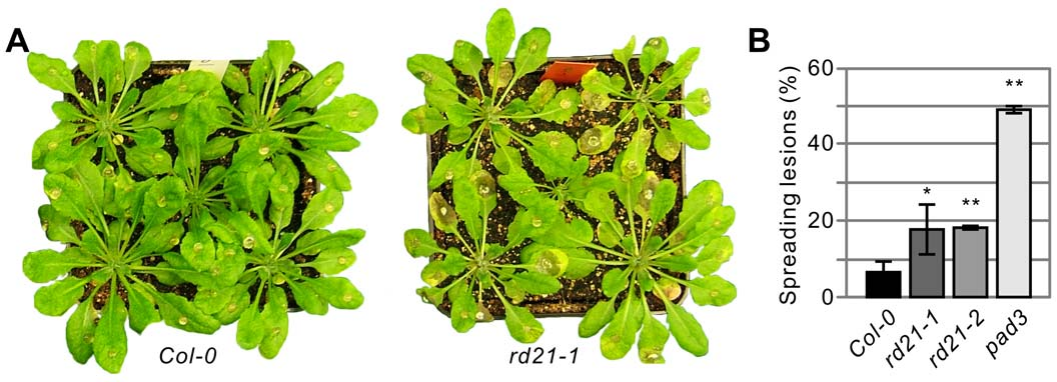
Mutant *rd21* lines have increased susceptibility for *Botrytis cinerea.* **A,** Infection of (mutant) Arabidopsis plants with *Bc*. Representative photographs were taken at six days after droplet inoculation. **B,** Frequency of lesion expansion. (Mutant) Arabidopsis plants were droplet-inoculated with spores of *Bc* and expansion of necrotic lesions was scored at 7 dpi. Error bars represent SD of three samples of 10 leaves each. This assay was repeated five times with similar results. *P*-values of Students *t*-test when compared to the Col-0 control are: *rd21-1*: 0.0414*; *rd21-2*: 0.0151**; and *pad3*: 0.008**.

To test if RD21 is involved in immunity against necrotrophic pathogens, we performed infection assays with the necrotrophic pathogen *Botrytis cinerea*. The *pad3* mutant, which is deficient in camalexin production [28], was included in these assays as a positive control for enhanced susceptibility. Leaves of (mutant) plants were inoculated with spore-containing droplets and scored for expanding lesions at 5–7 dpi. Importantly, both *rd21* mutants displayed more expanding lesions when compared to the wild-type control plants (**Figure 6A**). Quantification of the frequency of expanding lesions show a significantly enhanced frequency of lesion expansion for *rd21* mutant plants when compared to wild-type controls, though still reduced when compared to the *pad3* mutant (**Figure 6B**). These data demonstrate that *RD21* contributes to immunity to *B. cinerea* infection.

## Discussion

In this study, we set out to investigate the role of EPIC-C14 interactions in the *Hpa*-Arabidopsis pathosystem. Our journey identified Arabidopsis RD21 as the likely C14 ortholog, and EPIC homologs from *Hpa*. However, in contrast to earlier studies in the *Pinf*-solanaceae pathosystem, RD21 is conserved in *A. thaliana* and *rd21* mutants are not affected in compatible and incompatible interactions with *Hpa*, despite the evident lack of Cys protease activities in the leaf. However, the *rd21* knock-outs are significantly more susceptible for *Botrytis cinerea*, and not for virulent and avirulent strains of *Pto*DC3000. These data demonstrate that RD21 is involved in immunity to the necrotrophic fungal pathogen *Botrytis cinerea*.

### Are there EPIC-C14-like interactions in the Hpa-Arabidopsis pathosystem?

At this stage, it remains unknown if EPIC-C14-like interactions might exist in the *Hpa*-Arabidopsis pathosystem. We believe we have identified the right EPIC and C14 orthologs from *Hpa* and Arabidopsis, respectively. *Hpa*EPIC-B and *Hpa*EPIC-C are both expressed during infection and are likely secreted into the apoplast. Arabidopsis RD21 is the closest ortholog of C14 and is a very abundant Cys protease in leaves.

Despite the presence of EPIC and C14 orthologs, our further studies do not support the role of *Hpa*EPIC-RD21 interactions. First, *Hpa*EPIC-C lacks key cystatin-like motifs and is therefore unlikely to be an inhibitor of Cys proteases. The specific disruption of key cystatin motifs and the fact that this is conserved in different *Hpa* isolates is remarkable, and indicates that a different role has evolved for *Hpa*EPIC-C. *Hpa*EPIC-B, however, carries all essential cystatin-like motifs and is therefore a likely Cys protease inhibitor.

A second aspect is that we expected the *Hpa*-Arabidopsis pathosystem to be co-evolving in nature, similar to the *Pinf*-potato pathosystem, where the pattern of diversifying selection at the *C14* gene could have been caused by coevolution with the corresponding effectors [9]. We found, however, that *RD21* is conserved in *A. thaliana*. This situation is similar to that of C14 in tomato, which is nevertheless targeted by *Pinf*EPICs [9]. Diversifying selection may not be essential for antagonistic protein-protein interactions at the plant-pathogen interface.

There are different explanations for the observed conservative selection of RD21 in *A. thaliana*. Nucleotide diversity in *A. thaliana* is generally low and it has been suggested that this overall pattern is due to purifying selection acting on the *A. thaliana* genome and/or a recent population expansion [29]–[32]. However, the pattern of strong protein conservation we observed for the *RD21A* gene is slightly stronger than this overall pattern in the species, suggesting that *RD21A* is under purifying selection. Some caution is needed interpreting these data since we did not calculate the variation in the genomic background for the same dataset. Nevertheless, we can conclude that selective pressures acting on potato *C14* and Arabidopsis *RD21A* differ substantially. These data indicate that RD21 is strongly conserved because of a pivotal process in plants, that remains to be identified. Meanwhile, it is possible that RD21 is targeted by pathogen-derived effectors, but environmental, spatial or temporal conditions of host-pathogen interactions do not promote rapid diversification of this protease beyond the internal constraints on RD21 function.

A third distinct difference to the EPIC-C14 interaction is that the *rd21* mutant plants behave indistinguishable from wild-type plants in compatible and incompatible interactions with *Hpa*. This is in strong contrast to *C14*-silenced *N. benthamiana* plants, which are significantly more susceptible for infection by *P. infestans* [9]. The absence of a phenotype in *Hpa* assays is remarkable since the *rd21* mutant plants are clearly impaired in Cys protease activities. However, the absence of phenotypes for *rd21* mutants does not imply that the *Hpa*EPIC-RD21 interactions do not exist. In fact, effective inhibition of RD21 by *Hpa*EPICs or other ways of manipulation might rather make *Hpa* insensitive for the presence or absence of RD21. In this case, the role of RD21 would only be revealed in the *Hpa*-Arabidopsis interactions in the absence of *Hpa*-derived RD21 inhibitors. This paradox is similar to that of showing the role of PAMP-triggered immunity (PTI) when pathogens have evolved mechanism to suppress PTI [33].

There are two more issues unresolved for possible *Hpa*EPIC-RD21 interactions. First, it is unknown if *Hpa*EPICs and RD21 would colocalize in the apoplast. We presume that *Hpa*EPICs are secreted during infection into the apoplast. Arabidopsis RD21 is present in vesicles and the vacuole [23], [25], [34], and was not detected in leaf apoplastic fluids [35]. A second unresolved issue is whether or not *Hpa*EPICs physically interact with Arabidopsis RD21. We were unable to express and purify *Hpa*EPIC-B and *Hpa*EPIC-C proteins in sufficient amounts for inhibition and interaction studies. However, we did find that Arabidopsis RD21 can be inhibited by *Pinf*EPIC1 and *Pinf*EPIC2B (**Figure S8**), demonstrating that RD21 can be inhibited by EPIC-like proteins.

### The role of *rd21* in other pathosystems

The *rd21* mutants are unaffected in infection with virulent and avirulent *Pto*DC3000 strains. The absence of phenotypes for *rd21* mutants in these pathassays and during normal growth is remarkable given the fact that *rd21* mutants lack one of the major Cys protease activities in leaves. More specifically, since RD21 is an abundant vacuolar Cys protease [23], [25] and the vacuolar content is released during the hypersensitive response triggered by *Pto*DC000 producing avrRpm1 [26], we expected enhanced bacterial survival upon infection of *rd21* mutants, but this was not observed. However, in another study we have noticed that RD21 activity can be suppressed by *Pto*DC3000 during infection and we have identified an inhibitor of RD21 from the *Pto*DC3000 genome (Kaschani and Van der Hoorn, unpublished data). Thus, the effective production of inhibitors by *Pto*DC3000 may render these pathogens insensitive for host proteases. The fact that different pathogens produce Cys protease inhibitors indicates that host Cys proteases play a role during infection but more research will be required to test this hypothesis.

Interestingly, *rd21* mutant lines are more susceptible for *Botrytis cinerea*. Since *B. cinerea* is a necrotrophic fungal pathogen, it might be exposed to large amounts of vacuolar RD21 during host cell death. The sensitivity for host proteases correlates with the fact that the genome of *Botrytis cinerea* does not seem to encode obvious inhibitors of PLCPs [36], [37]. The absence of effective suppression mechanisms of host Cys proteases might originate from the fact that this pathogen has a broad, unspecialized host range and rather infects post-harvest fruits than healthy leaves [38].

In conclusion, our search for EPIC-C14-like interactions in the *Hpa*-Arabidopsis pathosystem let to a series of intriguing observations that illustrate differences rather than similarities between the *Hpa*-Arabidopsis and *Pinf*-solanaceae pathosystems. Although RD21 is the most likely ortholog of potato C14, it seems under purifying selection and the absence of RD21 does not affect *Hpa* infection, in contrast to C14 in the *Pinf*-solanaceae pathosystem. However, studying the role of Cys proteases is paradoxal when pathogens are armed with tools to suppress host protease activities. Our further studies nevertheless demonstrated that *rd21* mutants lack a major Cys protease and are more susceptible for infection with *Botrytis cinerea*.

## Materials and Methods

### Bioinformatics

To identify the ortholog of tomato C14 in the Arabidopsis genome, we performed a BLASTP search to the Arabidopsis TAIR10 database using the C14 protein sequence as a template. *E*-values of the BLAST search were used to define the closest ortholog to tomato C14. For phylogenetic assessment of the relationship of C14 to Arabidopsis proteases sequences of all Arabidopsis PLCPs and tomato C14, RCR3 and PIP1 were obtained from TAIR and GenBank (accession numbers: At1g02300, At3g48340, At3g48350, At1g06260, At3g43960, At1g09850, At1g20850, At1g29080, At1g29090, At1g47128, At2g21430, At2g27420, At2g34080, At3g19390, At3g19400, At3g45310, At3g49340, At3g54940, At4g11310, At4g11320, At4g16190, At4g23520, At4g35350, At4g36880, At4g39090, At5g43060, At5g45890, At5g50260, At5g60360 and At4g01610). Sequences were aligned using BioEdit 7.0.9 (Ibis Biosciences, Carlsbad, CA, USA) and Mesquite [39]. The first 66 amino acids at the N-terminus of the proteins, which exhibited only low homology between taxa, were removed from the alignment prior phylogenetic analyses. The phylogenetic relationships between the sequences were determined using PAUP v. 4.0b10 (Sinauer Associates). Arabidopsis Cathepsin B (CTB3) was used as outgroup to root the tree. Maximum parsimony and neighbor-joining methods were performed and these methods yielded similar topologies. To identify the ortholog of *Pinf*EPICs in the *Hpa* genome, we performed a BLASTP search on the genome database of *Hpa* isolate Emoy2 at the VBI Microbial Database using the *Pinf*EPICs as templates. For phylogenetic assessment of the relationship between the *Pinf* and *Hpa* EPICs amino acid sequences of these proteins were obtained from the VBI Microbial Database (605925, 603437, and 603438) and the GenBank (XP_002903480, XP_002903482, AAY21184, and XP_002908692). Sequence alignment and phylogenetic analyses on the full-length alignment were performed as described above. Cystatin from *Albugo laibachii* (GenBank accession number CCA25338) was used as outgroup to root the tree.

We performed population genetic analyses with *RD21* sequences from 80 Arabidopsis individuals [21]. The RD21 sequence of *A. lyrata* (NM_103612) was obtained from GenBank and used as outgroup. The standard summary statistics including π, divergence, Tajima's *D* (*D*_T_) and Fu and Li's *D* (*D*_F_) test statistics were calculated using DnaSP v. 5.10 [40]. The site frequency spectrum of mutations was determined using SITES [41].

### DNA work

For sequencing *Hpa*EPIC-encoding genes from other *Hpa* isolates, genomic DNA was isolated by grinding infected leaf material in liquid nitrogen with sand using mortar and pestle. The leaf material was mixed in 200 mM Tris pH 7.5, 250 mM NaCl, 0.5% SDS, 25 mM EDTA and centrifugated (5 minutes 16.000 g). DNA was precipitated from the supernatant by adding one volume isopropanol and centrifugating (5 minutes 16.000 g). The pellet was dissolved in 50 mM Tris pH8 EDTA by heating at 65°C for 5 minutes. *HpaEPIC* genes were amplified by PCR using primers that anneal in the 5′ and 3′ untranslated regions (**Table S1**). PCR fragments were sequenced from both sides. Forward and reverse sequences were aligned and observed nucleotide differences were verified in the trace data. For RT-PCR, RNA was isolated from *Hpa*-infected tissues at 5 dpi using RNeasy plant mini kit (Qiagen). Transcripts were amplified by semi-quantitative RT-PCR using primers summarized in Table S1.

### Protein work

DCG-04 labeling was performed on leaf extracts, generated by grinding an Arabidopsis leaf in 1 mL water in a 1.5 mL tube, and clearing by centrifugation for 5 minutes at 16.000 g. 100 µL of leaf extract was labeled with 2 µM DCG-04 in the presence of 1 mM DTT and 25 mM NaAc pH6, in a total volume of 500 µL for 5 hours at 22°C. Proteins were precipitated by adding 1 mL ice-cold acetone and the pellet was dissolved in 50 µL gel-loading buffer. Proteins were separated on 12% SDS-PAGE gels and transferred to PVDF membranes (Immobilon-P, Biorad), and detected using anti-FLAG antibody (Sigma-Aldrich), or streptavidin-HRP (Ultrasensitive, Sigma-Aldrich), or anti-RD21 antibody [23] and HRP-linked anti-rabbit antibody (Invitrogen).

### Disease assays

Plants were grown in a growth cabinet at 22°C at a 10-hour light regime and used for three different pathogen assays. Infection with *Hpa* by spray inoculation was performed on 3-week-old plants as described previously [42]. The numbers of spores at 7 dpi was counted by measuring the weight of infected plants, vortexing in water and counting released spores using a haemocytometer. Infection with *Bc* (strain iMi 169558) was performed on 5-week-old plants by inoculating each leaf with a 5 µL droplet of a 10^6^ spores/mL LB spore suspension. The plants were kept at high humidity and the percentage of leaves showing spreading lesions was scored at 5–7 dpi. Infections with *Pst*DC3000 were performed by spray inoculation of 5-week-old plants with 5×10^8^ bacteria in 10 mM MgCl_2_ 0.05% Silwet L77 and bacterial growth was measured at 0 and 3 dpi by plating dilution series of extracts on solid selection medium and counting colonies.

## Supporting Information

Figure S1 **Expression of** ***RD21A*** **and** ***RD21B*** **during Arabidopsis development. Data were extracted from Genevestigator.**
(PDF)

Figure S2 **Overview of polymorphisms in the** ***RD21A*** **gene in 80 individuals of** ***A. thaliana*** **.** The amino acid sequence at polymorphic positions is given in the one-letter code of amino acids using the ecotype Col-0 as a reference. Identical amino acids encoded by identical codons are indicated with dots, synonymous polymorphisms are labelled with grey boxes and nonsynonymous polymorphisms are labelled with red boxes. Polymorphic positions in the same codon are indicated by black lines on top.
(PDF)

Figure S3 **The site frequency spectrum of 80** ***RD21A*** **alleles reveals an excess of polymorphisms in low frequency.** The frequency of synonymous (light blue) and nonsynonymous (dark blue) mutations occurring at a certain number in the dataset (mutational class) is blotted for each mutational class. The red line indicates the expectation under complete neutrality.
(PDF)

Figure S4 **Polymorphism in RD21-encoding sequences of** ***A. lyrata*** **and** ***thaliana*** **.** Single nucleotide polymorphisms are indicated in grey on sequences of *thaliana* Col-0 (black) and *lyrata* (gi|297852301, red). Variant codons encode identical amino acids (light grey), similar amino acids (dark grey) and non-similar amino acids (red). The protease domain is printed in bold amino acids.
(PDF)

Figure S5 **Sequences and alignments of** ***Hpa*** **EPIC-B from various isolates.**
(PDF)

Figure S6 **Sequences and alignments of** ***Hpa*** **EPIC-C from various isolates.**
(PDF)

Figure S7 **Both** ***rd21-1*** **and** ***rd21-2*** **are null mutants.** Leaf extracts of Col-0 and *rd21* mutant plants were labelled with DCG-04 in the presence or absence of an excess E-64 and separated on protein gels. Proteins were detected with RD21 antibody (**A**) and streptavidin-HRP (**B**), and coomassie staining (**C**).
(PDF)

Figure S8 ***Pinf*** **EPICs inhibit Arabidopsis RD21.** *Nicotiana benthamiana* leaf extracts transiently overexpressing Arabidopsis RD21 (by agroinfiltration) were preincubated (30 min) with 40 µM E-64 or with 1.2 µM PinfEPIC1, *Pinf*EPIC2B (Tian et al., 2007) or chicken Cystatin (Sigma) and then labelled with 4 µM fluorescent DCG-04 for 2 hours. Proteins were separated on protein gels and fluorescent proteins were detected using a fluorescence scanner.
(PDF)

Table S1 Sequences of primers used in this study.
(PDF)
