# Supplementary material for: A Role in Immunity for Arabidopsis Cysteine Protease RD21, the Ortholog of the Tomato Immune Protease C14
Source: PLoS One. 2012 Jan 6;7(1):e29317. doi: 10.1371/journal.pone.0029317 (PMC3253073; doi:10.1371/journal.pone.0029317)
Supplement: Figure S1 — Expression of RD21A and RD21B during Arabidopsis development. Data were extracted from Genevestigator. (PDF) [file pone.0029317.s001.pdf]

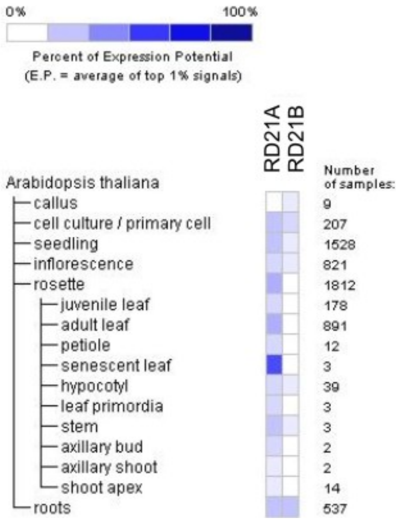

Figure S1. Expression of *RD21A* and *RD21B* during Arabidopsis development. Data were extracted from Genevestigator.
