# Supplementary material for: A Role in Immunity for Arabidopsis Cysteine Protease RD21, the Ortholog of the Tomato Immune Protease C14
Source: PLoS One. 2012 Jan 6;7(1):e29317. doi: 10.1371/journal.pone.0029317 (PMC3253073; doi:10.1371/journal.pone.0029317)
Supplement: Figure S2 — Overview of polymorphisms in the RD21A gene in 80 individuals of A. thaliana . The amino acid sequence at polymorphic positions is given in the one-letter code of amino acids using the ecotype Col-0 as a reference. Identical amino acids encoded by identical codons are indicated with dots, synonymous polymorphisms are labelled with grey boxes and nonsynonymous polymorphisms are labelled with red boxes. Polymorphic positions in the same codon are indicated by black lines on top. (PDF) [file pone.0029317.s002.pdf]

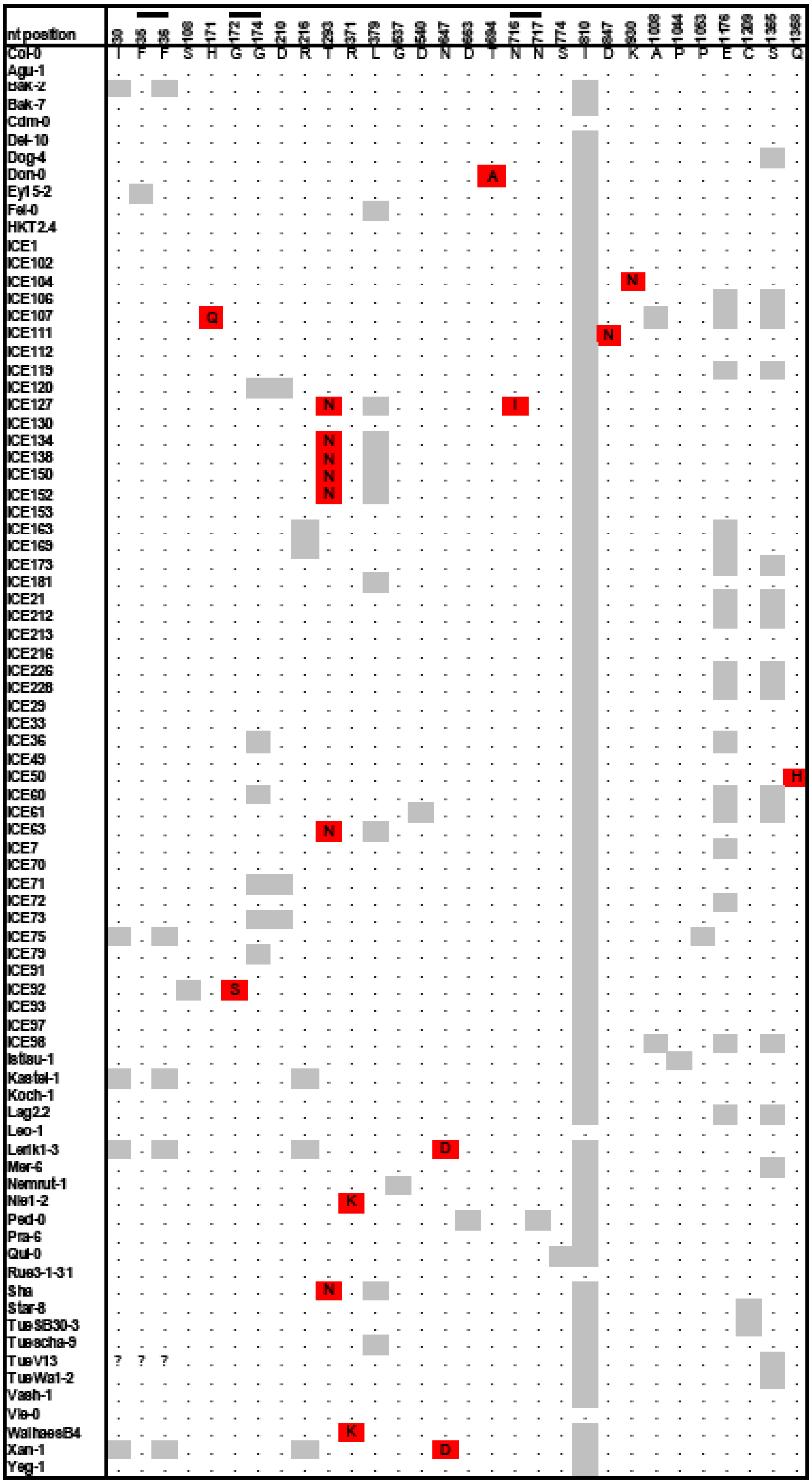

Figure S2. Overview of polymorphisms in the *RD21A* gene in 80 individuals of *A. thaliana*. The amino acid sequence at polymorphic positions is given in the one-letter code of amino acids using the ecotype Col-0 as a reference. Identical amino acids encoded by identical codons are indicated with dots, synonymous polymorphisms are labelled with grey boxes and nonsynonymous polymorphisms are labelled with red boxes. Polymorphic positions in the same codon are indicated by black lines on top.
