# Supplementary material for: A Role in Immunity for Arabidopsis Cysteine Protease RD21, the Ortholog of the Tomato Immune Protease C14
Source: PLoS One. 2012 Jan 6;7(1):e29317. doi: 10.1371/journal.pone.0029317 (PMC3253073; doi:10.1371/journal.pone.0029317)
Supplement: Figure S3 — The site frequency spectrum of 80 RD21A alleles reveals an excess of polymorphisms in low frequency. The frequency of synonymous (light blue) and nonsynonymous (dark blue) mutations occurring at a certain number in the dataset (mutational class) is blotted for each mutational class. The red line indicates the expectation under complete neutrality. (PDF) [file pone.0029317.s003.pdf]

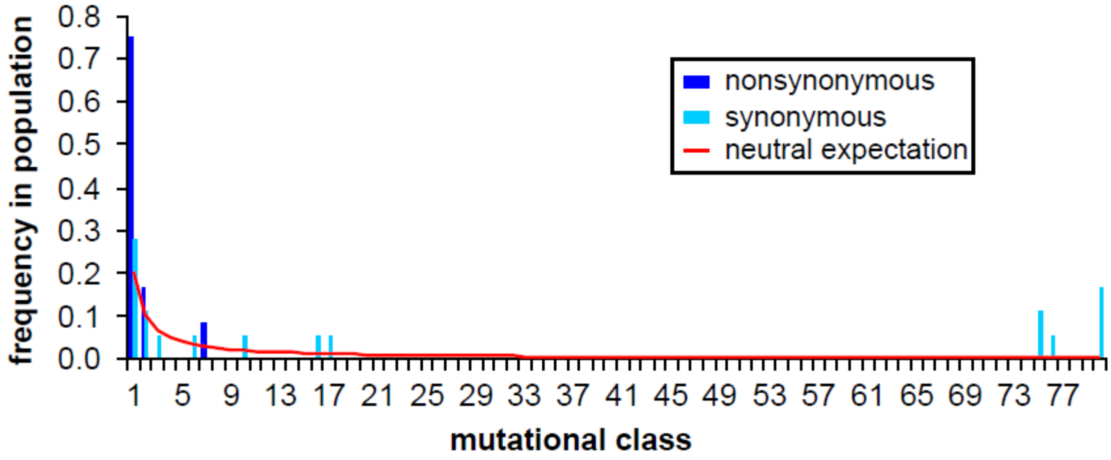

Figure S3. The site frequency spectrum of 80 *RD21A* alleles reveals an excess of polymorphisms in low frequency. The frequency of synonymous (light blue) and nonsynonymous (dark blue) mutations occurring at a certain number in the dataset (mutational class) is blotted for each mutational class. The red line indicates the expectation under complete neutrality.
