# Supplementary material for: A Role in Immunity for Arabidopsis Cysteine Protease RD21, the Ortholog of the Tomato Immune Protease C14
Source: PLoS One. 2012 Jan 6;7(1):e29317. doi: 10.1371/journal.pone.0029317 (PMC3253073; doi:10.1371/journal.pone.0029317)
Supplement: Figure S4 — Polymorphism in RD21-encoding sequences of A. lyrata and thaliana . Single nucleotide polymorphisms are indicated in grey on sequences of thaliana Col-0 (black) and lyrata (gi|297852301, red). Variant codons encode identical amino acids (light grey), similar amino acids (dark grey) and non-similar amino acids (red). The protease domain is printed in bold amino acids. (PDF) [file pone.0029317.s004.pdf]

```
   1 M  G  F  L  K  P  T  M  AV II L  FF L  A  M  V  A  V  SA S
   1 ATGGGGTTCCTTAAGCCAACCATGGCGATTCTTTTTCTAGCGATGGTCGCCGTTTCATCA
                          ATGGTGATCCTTTTCCTAGCGATGGTCGCCGTTGCATCA
  21 A  V  D  M  SS I  I  S  Y  D  E  K  HH G  V  S  T  T  G  G
  61 GCCGTGGACATGTCAATCATCTCCTACGACGAGAAACATGGCGTCTCCACCACCGGTGGC
     GCCGTGGACATGTCCATCATCTCCTACGACGAGAAACACGGCGTCTCCACCACCGGTGGC
  41 R  S  ED AA E  V  M  S  I  Y  E  AA W  L  V  K  H  G  K  A
 121 CGTAGCGAAGCCGAGGTTATGAGTATCTACGAGGCATGGTTGGTGAAACACGGCAAGGCT
     CGTAGCGACGCTGAGGTTATGAGTATCTACGAGGCGTGGTTGGTGAAACACGGCAAGGCT
  61 Q  SN Q  N  S  L  V  E  K  D  R  RR F  E  I  F  K  D  N  L
 181 CAGAGCCAGAACTCTCTTGTTGAGAAAGATCGACGGTTCGAGATCTTTAAAGACAATCTT
     CAGAATCAGAACTCTCTTGTTGAGAAAGATCGACGTTTCGAGATCTTTAAAGACAATCTT
  81 R  F  VI DD ED H  N  EK K  N  L  S  Y  R  L  G  L  T  RR F
 241 CGTTTCGTCGATGAACATAACGAGAAGAATCTTAGTTATAGATTGGGTTTGACTCGTTTT
     CGTTTCATCGACGATCATAACAAGAAGAATCTTAGTTATAGATTGGGTTTGACTCGGTTT
 101 A  D  L  T  N  D  E  Y  R  S  K  YY L  G  A  K  M  E  K  K
 301 GCGGATCTTACTAACGATGAGTATAGATCCAAGTATCTTGGAGCTAAGATGGAGAAGAAA
     GCGGATCTTACTAACGATGAGTATAGATCCAAGTACCTTGGAGCTAAGATGGAGAAGAAA
 121 G  E  R  R  T  S  LQ RR Y  E  A  R  V  G  D  E  L  P  E  S
 361 GGTGAGAGAAGGACTAGCCTACGGTACGAGGCTCGTGTCGGTGATGAGCTACCGGAGTCT
     GGTGAGAGAAGGACTAGCCAACGCTACGAGGCTCGTGTCGGTGATGAGCTACCGGAGTCT
 141 I  D  W  R  K  K  G  A  V  A  E  V  K  D  Q  G  GS C  G  S
 421 ATTGACTGGAGGAAGAAAGGAGCCGTGGCTGAGGTCAAAGATCAGGGTGGTTGCGGGAGT
     ATTGACTGGAGGAAGAAAGGAGCCGTGGCTGAGGTCAAAGATCAGGGTAGTTGCGGGAGT
 161 C  W  AA FF S  T  I  G  A  V  E  GG I  N  Q  I  V  TT G  DD
 481 TGTTGGGCGTTTTCAACCATTGGAGCAGTGGAGGGAATAAACCAGATCGTAACCGGAGAC
     TGTTGGGCATTCTCAACCATTGGAGCAGTGGAGGGGATAAACCAGATCGTAACAGGAGAT
 181 LL I  T  L  S  E  Q  E  L  V  D  C  D  T  S  Y  NN E  G  C
 541 CTAATAACCTTGTCTGAACAAGAGTTGGTCGATTGTGACACTTCATACAACGAAGGTTGT
     TTAATAACCTTGTCTGAACAAGAGTTGGTCGATTGTGACACTTCATACAATGAAGGTTGT
 201 N  GG G  LL M  D  Y  A  F  EE F  I  I  K  N  G  G  I  D  T
 601 AACGGAGGTCTTATGGACTATGCTTTTGAGTTCATTATCAAGAATGGTGGAATCGATACA
     AACGGTGGTCTCATGGACTATGCTTTTGAATTCATTATCAAGAATGGTGGAATCGATACA
 221 D  K  D  Y  P  Y  K  G  VV D  G  T  C  D  Q  I  R  K  N  A
 661 GACAAAGATTATCCTTACAAGGGTGTTGATGGAACTTGTGACCAGATCAGGAAAAACGCT
     GACAAAGATTATCCTTACAAGGGTGTCGATGGAACTTGTGACCAGATCAGGAAAAACGCT
 241 K  V  V  TT II D  S  Y  E  D  V  PP T  Y  S  E  E  SS L  K
 721 AAAGTTGTCACTATCGATTCATATGAGGATGTTCCAACTTACAGCGAGGAATCGTTGAAG
     AAAGTTGTCACAATTGATTCATATGAGGATGTTCCCACTTACAGCGAGGAATCATTGAAG
 261 K  A  V  A  H  Q  P  IV S  IV A  I  E  A  G  G  R  A  F  Q
 781 AAAGCAGTTGCTCATCAACCCATTAGCATTGCCATTGAGGCTGGTGGTCGTGCGTTCCAG
     AAAGCAGTTGCTCATCAACCCGTTAGCGTCGCCATTGAGGCTGGTGGTCGTGCGTTCCAG
 281 L  Y  D  S  G  I  F  D  G  ST C  G  T  Q  L  D  H  G  V  V
 841 CTCTATGATTCGGGTATATTTGATGGAAGTTGTGGAACACAACTAGACCACGGAGTTGTG
     CTCTATGATTCGGGTATATTTGATGGAACTTGTGGAACACAACTAGACCACGGAGTTGTG
 301 A  V  G  Y  G  TT E  N  G  K  DD Y  W  I  V  R  N  S  W  G
 901 GCGGTTGGATACGGAACTGAGAACGGCAAAGATTACTGGATTGTGAGAAACTCATGGGGT
     GCGGTTGGATACGGAACCGAGAACGGCAAAGACTACTGGATTGTGAGAAACTCATGGGGT
 321 K  SS W  G  E  S  G  Y  LL RK M  A  R  N  II A  S  S  SS G
 961 AAAAGCTGGGGAGAGAGTGGATACCTAAGGATGGCGCGTAACATTGCGTCTTCATCAGGA
     AAAAGTTGGGGAGAGAGTGGATACTTAAAGATGGCGCGTAACATCGCGTCTTCATCTGGA
 341 K  C  G  I  A  I  E  P  S  Y  PP II K  N  G  E  N  PP P  N
1021 AAATGTGGAATCGCGATTGAACCTTCATACCCGATAAAGAATGGCGAAAACCCGCCAAAC
     AAATGTGGAATCGCGATTGAACCTTCATACCCAATCAAGAATGGCGAAAACCCACCAAAC
 361 P  G  P  S  P  P  S  P  I  K  P  P  T  Q  C  D  S  Y  Y  T
1081 CCGGGACCTTCACCTCCATCTCCCATCAAGCCTCCAACCCAATGTGACAGTTACTACACT
     CCGGGACCTTCACCTCCATCTCCCATCAAGCCTCCAACCCAATGTGACAGTTACTACACT
 381 C  P  E  S  N  T  C  C  C  LL F  E  Y  G  K  Y  C  F  A  W
1141 TGTCCTGAGAGCAACACTTGTTGTTGTCTGTTTGAGTATGGCAAGTATTGCTTTGCTTGG
     TGTCCTGAGAGCAACACTTGTTGTTGTCTCTTTGAGTATGGCAAGTATTGCTTTGCTTGG
 401 G  C  C  P  L  E  A  A  T  C  C  D  D  N  Y  S  C  C  P  H
1201 GGATGTTGCCCACTAGAAGCAGCCACTTGCTGTGATGACAACTATAGTTGCTGCCCTCAC
     GGATGTTGCCCACTAGAAGCAGCCACTTGCTGTGATGACAACTATAGTTGCTGCCCTCAC
 421 EE Y  P  V  C  D  L  D  QQ G  T  C  LL L  S  K  N  S  P  F
1261 GAGTACCCGGTTTGTGACCTTGATCAAGGAACCTGTTTATTGAGCAAGAACAGTCCATTT
     GAATACCCGGTTTGTGACCTTGATCAGGGAACCTGTTTGTTGAGCAAGAACAGTCCATTT
 441 S  V  K  A  L  K  R  K  P  A  T  P  F  W  S  Q  G  R  K  N
1321 AGTGTTAAGGCCTTAAAGCGTAAACCCGCAACGCCATTCTGGTCACAAGGCAGAAAGAAC
     AGTGTTAAGGCCTTAAAGCGTAAACCCGCAACGCCATTCTGGTCACAAGGCAGAAAGAAC
 461 I  A  *
1381 ATTGCCTAA
     ATTGCCTAA
```

Figure S4. Polymorphism in RD21-encoding sequences of *A. lyrata* and *thaliana*. Single nucleotide polymorphisms are indicated in grey on sequences of *thaliana* Col-0 (black) and *lyrata* (gi|297852301, red). Variant codons encode identical amino acids (light grey), similar amino acids (dark grey) and non-similar amino acids (red). The protease domain is printed in bold amino acids.
