# Supplementary material for: A Role in Immunity for Arabidopsis Cysteine Protease RD21, the Ortholog of the Tomato Immune Protease C14
Source: PLoS One. 2012 Jan 6;7(1):e29317. doi: 10.1371/journal.pone.0029317 (PMC3253073; doi:10.1371/journal.pone.0029317)
Supplement: Figure S5 — Sequences and alignments of Hpa EPIC-B from various isolates. (PDF) [file pone.0029317.s005.pdf]

Genomic DNA sequences of *Hpa*EPIC-B:

```
>EMOYB 603438 8185
ATGACGTCCTTCCGCTCGGTTGCCCTTCTCGCTGGACTGGCTCTATCTTCGACGGATGTCCAAGGGCTACTACCAGAGCATATGCCTGACGTGATAGTGGGTGGCTACAGCACGCCACGAACTATGACCCTGAACGAG
GTCGCGTTTCTGACAACAACTGCATGCCATCCAAGCCTGTACACTGCGGGCGTAACCAGCCGGATCTGCTTTACGGAATTCGGCTCGATTCAGTCGCAAGCTGTGTCTGGTACGAACGACATGTTCATGGTGAAAGGC
TGCCCTGTGAACAGGGACGAACACTTGGGCTACTGCCGCGATGGCGTTTGTTCCACGACCTCGACCTACGAAGTCATCATCTACTCGCAGGTGTGGACGAACACGGTCAACGTGACGTCGGTCAGGGAGGTAAATGCA
GGTGAATCTACTCCATACCCAACGACACTGTAGCTTTCAACTCTATACTTCTAACCTGTTCCGGTGAAAATAGAGGTTGACGAACTCGTCACTTTTGAAAGTTGTGTTGATCTGTTGTGGACTCGGAACCTTTGCTGA
TTTATTGAAGGATGGAGATAGGAACTTTGTTGAGGGCATATGCCGTGTTCTAGAGCATACACATAGTGCCACATGTATCTGGTCTACGTCTGCATGCGAAAGAAGCACCCCAGCATGCAACTTGTATGA
>NOCOB
ATGACGTCCTTCCGCTCGGTTGCCCTTCTCGCTGGACTGGCTCTATCTTCGACGGATGTCCAAGGGCTACTACCAGAGCATATGCCTGACGTGATAGTGGGTGGCTACAGCACGCCACGAACTATGACCCTGAACGAG
GTCGCGTTTCTGACAACAACTGCATGCCATCCAAGCCTGTACACTGCGGGCGTAACCAGCCGGATCTGCTTTACGGAATTCGGCTCGATTCAGTCGCAAGCTGTGTCTGGTACGAACGACATGTTCATGGTGAAAGGC
TGCCCTGTGAACAGGGACGAACACTTGGGCTACTGCCGCGATGGCGTTTGTTCCACGACCTCGACCTACGAAGTCATCATCTACTCGCAGGTGTGGACGAACACGGTCAACGTGACGTCGGTCAGGGAGGTAAATGCA
GGTGAATCTACTCCATACCCAACGACACTGTAGCTTTCAACTCTATACTTCTAACCTGTTCCGGTGAAAATAGAGGTTGACGAACTCGTCACTTTTGAAAGTTGTGTTGATCTGTTGTGGACTCGGAACCTTTGCTGA
TTTATTGAAGGATGGAGATAGGAACTTTGTTGAGGGCATATGCCGTGTTCTAGAGCATACACATAGTGCCACATGTATCTGGTCTACGTCTGCATGCGAAAGAAGCACCCCAGCATGCAACTTGTATGA
>CALAB
ATGACGTCCTTCCGCTCGGTTGCCCTTCTCGCTGGACTGGCTCTATCTTCGACGGATGTCCAAGGGCTACTACCAGAGCATATGCCTGACGTGATAGTGGGTGGCTACAGCACGCCACGAACTATGACCCTGAACGAG
GTCGCGTTTCTGACAACAACTGCATGCCATCCAAGCCTGTACACTGCGGGCGTAACCAGCCGGATCTGCTTTACGGAATTCGGCTCGATTCAGTCGCAAGCTGTGTCTGGTACGAACGACATGTTCATGGTGAAAGGC
TGCCCTGTGAACAGGGACGAACACTTGGGCTACTGCCGCGATGGCGTTTGTTCCACGACCTCGACCTACGAAGTCATCATCTACTCGCAGGTGTGGACGAACACGGTCAACGTGACGTCGaTCAGGGAGGTAAATGCA
GGTGAATCTACTCCATACCCAACGACACTGTAGCTTTCAACTCTATACTTCTAACCTGTTCCGGTGAAAATAGAGGTTGACGAACTCGTCACTTTTGAAAGTTGTGTTGATCTGTTGTGGACTCGGAACCTTTGCTGA
TTTATTGAAGGATGGAGATAGGAACTTTGTTGAGGGCATATGCCGTGTTCTAGAGCATACACATAGTGCCACATGTATCTGGTCTACGTCTGCATGCGAAAGAAGCACCCCAGCATGCAACTTGTATGA
>EMWAB
ATGACGTCCTTCCGCTCGGTTGCCCTTCTCGCTGGACTGGCTCTATCTTCGACGGATGTCCAAGGGCTACTACCAGAGCATATGCCTGACGTGATAGTGGGTGGCTACAGCACGCCACGAACTATGACCCTGAACGAG
GTCGCGTTTCTGACAACAACTGCATGCCATCCAAGCCTGTACACTGCGGGCGTAACCAGCCGGATCTGCTTTACGGAATTCGGCTCGATTCAGTCGCAAGCTGTGTCTGGTACGAACGACATGTTCATGGTGAAAGGC
TGCCCTGTGAACAGGGACGAACACTTGGGCTACTGCCGCGATGGCGTTTGTTCCACGACCTCGACCTACGAAGTCATCATCTACTCGCAGGTGTGGACGAACACGGTCAACGTGACGTCGGTCAGGGAGGTAAATGCA
GGTGAATCTACTCCATACCCAACGACACTGTAGCTTTCAACTCTATACTTCTAACCTGTTCCGGTGAAAATAGAGGTTGACGAACTCGTCACTTTTGAAAGTTGTGTTGATCTGTTGTGGACTCGGAACCTTTGCTGA
TTTATTGAAGGATGGAGATAGGAACTTTGTTGAGGGCATATGCCGTGTTCTAGAGCATACACATAGTGCCACATGTATCTGGTCTACGTCTGCATGCGAAAGAAGCACCCCAGCATGCAACTTGTATGA
>WACOB (incomplete)
TACAGCACGCCACGAACTATGACCCTGAACGAGGTCGCGTTTCTGACAACAACTGCATGCCATCCAAGCCTGTACACTGCGGGCGTAACCAGCCGGATCTGCTTTACGGAATTCGGCTCGATTCAGTCGCAAGCTGTG
TCTGGTACGAACGACATGTTCATGGTGAAAGGCTGCCCTGTGAACAGGGACGAACACTTGGGCTACTGCCGCGATGGCGTTTGTTCCACGACCTCGACCTACGAAGTCATCATCTACTCGCAGGTGTGGACGAACACG
GTCAACGTGACGTCGaTCAGGGAGGTAAATGCAGGTGAATCTACTCCATACCCAACGACACTGTAGCTTTCAACTCTATACTTCTAACCTGTTCCGGTGAAAATAGAGGTTGACGAACTCGTCACTTTTGAAAGTTGT
GTTGATCTGTTGTGGACTCGGAACCTTTGCTGATTTATTGAAGGATGGAGATAGGAACTTTGTTGAGGGCATATGCCGTGTTCTAGAGCATACACATAGTGCCACATGTATCTGGTCTAC
>MAKSB (incomplete)
ACCAGAGCATATGCCTGACGTGATAGTGGGTGGCTACAGCACGCCACGAACTATGACCCTGAACGAGGTCGCGTTTCTGACAACAACTGCATGCCATCCAAGCCTGTACACTGCGGGCGTAACCAGCCGGATCTGCTT
TACGGAATTCGGCTCGATTCAGTCGCAAGCTGTGTCTGGTACGAACGACATGTTCATGGTGAAAGGCTGCCCTGTGAACAGGGACGAACACTTGGGCTACTGCCGCGATGGCGTTTGTTCCACGACCTCGACCTACGA
AGTCATCATCTACTCGCAGGTGTGGACGAACACGGTCAACGTGACGTCGaTCAGGGAGGTAAATGCAGGTGAATCTACTCCATACCCAACGACACTGTAGCTTTCAACTCTATACTTCTAACCTGTTCCGGTGAAAAT
AGAGGTTGACGAACTCGTCACTTTTGAAAGTTGTGTTGATCTGTTGTGGACTCGGAACCTTTGCTGATTTATTGAAGGATGGAGATAGGAACTTTGTTGAGGGCATATGCCGTGTTCTAGAGCATACACATAGTGCCA
CATGTATCTGGTCTACGTCTGCATGCGAAAGAAGCACCCCAGCATGCAACTTGTATGA
```

Nucleotide aligment of *Hpa*EPIC-B:

```
       1                                                                                                   100
EMOYB  ATGACGTCCT TCCGCTCGGT TGCCCTTCTC GCTGGACTGG CTCTATCTTC GACGGATGTC CAAGGGCTAC TACCAGAGCA TATGCCTGAC GTGATAGTGG
NOCOB  ATGACGTCCT TCCGCTCGGT TGCCCTTCTC GCTGGACTGG CTCTATCTTC GACGGATGTC CAAGGGCTAC TACCAGAGCA TATGCCTGAC GTGATAGTGG
EMWAB  ATGACGTCCT TCCGCTCGGT TGCCCTTCTC GCTGGACTGG CTCTATCTTC GACGGATGTC CAAGGGCTAC TACCAGAGCA TATGCCTGAC GTGATAGTGG
CALAB  ATGACGTCCT TCCGCTCGGT TGCCCTTCTC GCTGGACTGG CTCTATCTTC GACGGATGTC CAAGGGCTAC TACCAGAGCA TATGCCTGAC GTGATAGTGG
MAKSB                                                                            ACCAGAGCA TATGCCTGAC GTGATAGTGG
WACOB

       101                                                                                                 200
EMOYB  GTGGCTACAG CACGCCACGA ACTATGACCC TGAACGAGGT CGCGTTTCTG ACAACAACTG CATGCCATCC AAGCCTGTAC ACTGCGGGCG TAACCAGCCG
NOCOB  GTGGCTACAG CACGCCACGA ACTATGACCC TGAACGAGGT CGCGTTTCTG ACAACAACTG CATGCCATCC AAGCCTGTAC ACTGCGGGCG TAACCAGCCG
EMWAB  GTGGCTACAG CACGCCACGA ACTATGACCC TGAACGAGGT CGCGTTTCTG ACAACAACTG CATGCCATCC AAGCCTGTAC ACTGCGGGCG TAACCAGCCG
CALAB  GTGGCTACAG CACGCCACGA ACTATGACCC TGAACGAGGT CGCGTTTCTG ACAACAACTG CATGCCATCC AAGCCTGTAC ACTGCGGGCG TAACCAGCCG
MAKSB  GTGGCTACAG CACGCCACGA ACTATGACCC TGAACGAGGT CGCGTTTCTG ACAACAACTG CATGCCATCC AAGCCTGTAC ACTGCGGGCG TAACCAGCCG
WACOB       TACAG CACGCCACGA ACTATGACCC TGAACGAGGT CGCGTTTCTG ACAACAACTG CATGCCATCC AAGCCTGTAC ACTGCGGGCG TAACCAGCCG

       201                                                                                                 300
EMOYB  GATCTGCTTT ACGGAATTCG GCTCGATTCA GTCGCAAGCT GTGTCTGGTA CGAACGACAT GTTCATGGTG AAAGGCTGCC CTGTGAACAG GGACGAACAC
NOCOB  GATCTGCTTT ACGGAATTCG GCTCGATTCA GTCGCAAGCT GTGTCTGGTA CGAACGACAT GTTCATGGTG AAAGGCTGCC CTGTGAACAG GGACGAACAC
EMWAB  GATCTGCTTT ACGGAATTCG GCTCGATTCA GTCGCAAGCT GTGTCTGGTA CGAACGACAT GTTCATGGTG AAAGGCTGCC CTGTGAACAG GGACGAACAC
CALAB  GATCTGCTTT ACGGAATTCG GCTCGATTCA GTCGCAAGCT GTGTCTGGTA CGAACGACAT GTTCATGGTG AAAGGCTGCC CTGTGAACAG GGACGAACAC
MAKSB  GATCTGCTTT ACGGAATTCG GCTCGATTCA GTCGCAAGCT GTGTCTGGTA CGAACGACAT GTTCATGGTG AAAGGCTGCC CTGTGAACAG GGACGAACAC
WACOB  GATCTGCTTT ACGGAATTCG GCTCGATTCA GTCGCAAGCT GTGTCTGGTA CGAACGACAT GTTCATGGTG AAAGGCTGCC CTGTGAACAG GGACGAACAC

       301                                                                                                 400
EMOYB  TTGGGCTACT GCCGCGATGG CGTTTGTTCC ACGACCTCGA CCTACGAAGT CATCATCTAC TCGCAGGTGT GGACGAACAC GGTCAACGTG ACGTCGGTCA
NOCOB  TTGGGCTACT GCCGCGATGG CGTTTGTTCC ACGACCTCGA CCTACGAAGT CATCATCTAC TCGCAGGTGT GGACGAACAC GGTCAACGTG ACGTCGGTCA
EMWAB  TTGGGCTACT GCCGCGATGG CGTTTGTTCC ACGACCTCGA CCTACGAAGT CATCATCTAC TCGCAGGTGT GGACGAACAC GGTCAACGTG ACGTCGGTCA
CALAB  TTGGGCTACT GCCGCGATGG CGTTTGTTCC ACGACCTCGA CCTACGAAGT CATCATCTAC TCGCAGGTGT GGACGAACAC GGTCAACGTG ACGTCGATCA
MAKSB  TTGGGCTACT GCCGCGATGG CGTTTGTTCC ACGACCTCGA CCTACGAAGT CATCATCTAC TCGCAGGTGT GGACGAACAC GGTCAACGTG ACGTCGATCA
WACOB  TTGGGCTACT GCCGCGATGG CGTTTGTTCC ACGACCTCGA CCTACGAAGT CATCATCTAC TCGCAGGTGT GGACGAACAC GGTCAACGTG ACGTCGATCA

       401                                                                                                 500
EMOYB  GGGAGGTAAA TGCAGGTGAA TCTACTCCAT ACCCAACGAC ACTGTAGCTT TCAACTCTAT ACTTCTAACC TGTTCCGGTG AAAATAGAGG TTGACGAACT
NOCOB  GGGAGGTAAA TGCAGGTGAA TCTACTCCAT ACCCAACGAC ACTGTAGCTT TCAACTCTAT ACTTCTAACC TGTTCCGGTG AAAATAGAGG TTGACGAACT
EMWAB  GGGAGGTAAA TGCAGGTGAA TCTACTCCAT ACCCAACGAC ACTGTAGCTT TCAACTCTAT ACTTCTAACC TGTTCCGGTG AAAATAGAGG TTGACGAACT
CALAB  GGGAGGTAAA TGCAGGTGAA TCTACTCCAT ACCCAACGAC ACTGTAGCTT TCAACTCTAT ACTTCTAACC TGTTCCGGTG AAAATAGAGG TTGACGAACT
MAKSB  GGGAGGTAAA TGCAGGTGAA TCTACTCCAT ACCCAACGAC ACTGTAGCTT TCAACTCTAT ACTTCTAACC TGTTCCGGTG AAAATAGAGG TTGACGAACT
WACOB  GGGAGGTAAA TGCAGGTGAA TCTACTCCAT ACCCAACGAC ACTGTAGCTT TCAACTCTAT ACTTCTAACC TGTTCCGGTG AAAATAGAGG TTGACGAACT

       501                                                                                                 600
EMOYB  CGTCACTTTT GAAAGTTGTG TTGATCTGTT GTGGACTCGG AACCTTTGCT GATTTATTGA AGGATGGAGA TAGGAACTTT GTTGAGGGCA TATGCCGTGT
NOCOB  CGTCACTTTT GAAAGTTGTG TTGATCTGTT GTGGACTCGG AACCTTTGCT GATTTATTGA AGGATGGAGA TAGGAACTTT GTTGAGGGCA TATGCCGTGT
EMWAB  CGTCACTTTT GAAAGTTGTG TTGATCTGTT GTGGACTCGG AACCTTTGCT GATTTATTGA AGGATGGAGA TAGGAACTTT GTTGAGGGCA TATGCCGTGT
CALAB  CGTCACTTTT GAAAGTTGTG TTGATCTGTT GTGGACTCGG AACCTTTGCT GATTTATTGA AGGATGGAGA TAGGAACTTT GTTGAGGGCA TATGCCGTGT
MAKSB  CGTCACTTTT GAAAGTTGTG TTGATCTGTT GTGGACTCGG AACCTTTGCT GATTTATTGA AGGATGGAGA TAGGAACTTT GTTGAGGGCA TATGCCGTGT
WACOB  CGTCACTTTT GAAAGTTGTG TTGATCTGTT GTGGACTCGG AACCTTTGCT GATTTATTGA AGGATGGAGA TAGGAACTTT GTTGAGGGCA TATGCCGTGT

       601                                                                               681
EMOYB  TCTAGAGCAT ACACATAGTG CCACATGTAT CTGGTCTACG TCTGCATGCG AAAGAAGCAC CCCAGCATGC AACTTGTATG A
NOCOB  TCTAGAGCAT ACACATAGTG CCACATGTAT CTGGTCTACG TCTGCATGCG AAAGAAGCAC CCCAGCATGC AACTTGTATG A
EMWAB  TCTAGAGCAT ACACATAGTG CCACATGTAT CTGGTCTACG TCTGCATGCG AAAGAAGCAC CCCAGCATGC AACTTGTATG A
CALAB  TCTAGAGCAT ACACATAGTG CCACATGTAT CTGGTCTACG TCTGCATGCG AAAGAAGCAC CCCAGCATGC AACTTGTATG A
MAKSB  TCTAGAGCAT ACACATAGTG CCACATGTAT CTGGTCTACG TCTGCATGCG AAAGAAGCAC CCCAGCATGC AACTTGTATG A
WACOB  TCTAGAGCAT ACACATAGTG CCACATGTAT CTGGTCTAC
```

Protein alignment of *Hpa*EPIC-B:

```
       1                                                                                                   100
EMOYB  MTSFRSVALL AGLALSSTDV QGLLPEHMPD VIVGGYSTPR TMTLNEVAFL TTTACHPSLY TAGVTSRICF TEFGSIQSQA VSGTNDMFMV KGCPVNRDEH
NOCOB  MTSFRSVALL AGLALSSTDV QGLLPEHMPD VIVGGYSTPR TMTLNEVAFL TTTACHPSLY TAGVTSRICF TEFGSIQSQA VSGTNDMFMV KGCPVNRDEH
EMWAB  MTSFRSVALL AGLALSSTDV QGLLPEHMPD VIVGGYSTPR TMTLNEVAFL TTTACHPSLY TAGVTSRICF TEFGSIQSQA VSGTNDMFMV KGCPVNRDEH
CALAB  MTSFRSVALL AGLALSSTDV QGLLPEHMPD VIVGGYSTPR TMTLNEVAFL TTTACHPSLY TAGVTSRICF TEFGSIQSQA VSGTNDMFMV KGCPVNRDEH
MAKSB                            PEHMPD VIVGGYSTPR TMTLNEVAFL TTTACHPSLY TAGVTSRICF TEFGSIQSQA VSGTNDMFMV KGCPVNRDEH
WACOB                                        YSTPR TMTLNEVAFL TTTACHPSLY TAGVTSRICF TEFGSIQSQA VSGTNDMFMV KGCPVNRDEH

       101                                                   155
EMOYB  LGYCRDGVCS TTSTYEVIIY SQVWTNTVNV TSVRECHMYL VYVCMRKKHP SMQLV
NOCOB  LGYCRDGVCS TTSTYEVIIY SQVWTNTVNV TSVRECHMYL VYVCMRKKHP SMQLV
EMWAB  LGYCRDGVCS TTSTYEVIIY SQVWTNTVNV TSVRECHMYL VYVCMRKKHP SMQLV
CALAB  LGYCRDGVCS TTSTYEVIIY SQVWTNTVNV TSIRECHMYL VYVCMRKKHP SMQLV
MAKSB  LGYCRDGVCS TTSTYEVIIY SQVWTNTVNV TSIRECHMYL VYVCMRKKHP SMQLV
WACOB  LGYCRDGVCS TTSTYEVIIY SQVWTNTVNV TSIRECHMYL VY
```

Figure S5. Sequences and alignments of *Hpa*EPIC-B from various isolates.
