# Supplementary material for: A Role in Immunity for Arabidopsis Cysteine Protease RD21, the Ortholog of the Tomato Immune Protease C14
Source: PLoS One. 2012 Jan 6;7(1):e29317. doi: 10.1371/journal.pone.0029317 (PMC3253073; doi:10.1371/journal.pone.0029317)
Supplement: Figure S6 — Sequences and alignments of Hpa EPIC-C from various isolates. (PDF) [file pone.0029317.s006.pdf]

Genomic DNA sequences of *Hpa*EPIC-C:

```
>EMOYC 603437
ATGATGTCCTTCCGCACGGCTGCTCTATTCGCTGGGCTGGCTCTAACTTCGTCGGCTGCTCAAGAGCAACAGCCTGTTCATGATCTAGGGTTGAACGCCTACGACCAGGCACGAGATGTGACCCTGAACGAGGTCGCG
TTCCTGACAACGACAGCATGTCATCCAAGCCTGTACAATGCGGATGTGACCAGCCGGGTCTGCTTCACGGAATTTACAACGGTGACGACGCAAACAAGTGGGGGTGGTACATACTACAAGTTTCAAGTGAAGGGCTGC
CCTGTGGACACGGAAAAACAGTTGGGCTACTGCCGCGAGGGTGCATGCTCGACGACCTCGCTCTACGAAGTCGCCATCTACTCGCAGCCGCGGACGAGCGCAGTCTTTCTGACGTCTATCAAGGAGGTAGTTTAA
>EMWAC
ATGATGTCCTTCCGCACGGCTGCTCTATTCGCTGGGCTGGCTCTAACTTCGTCGGCTGCTCAAGAGCAACAGCCTGTTCATGATCTAGGGTTGAACGCCTACGACCAGGCACGAGATGTGACCCTGAACGAGGTCGCG
TTCCTGACAACGACAGCATGTCATCCAAGCCTGTACAATGCGGATGTGACCAGCCGGGTCTGCTTCACGGAATTTACAACGGTGACGACGCAAACAAGTGGGGGTGGTACATACTACAAGTTTCAAGTGAAGGGCTGC
CCTGTGGACACGGAAAAACAGTTGGGCTACTGCCGCGAGGGTGCATGCTCGACGACCTCGCTCTACGAAGTCGCCATCTACTCGCAGCCGCGGACGAGCGCAGTCTTTCTGACGTCTATCAAGGAGGTAGTTTAA
>NOCOC
ATGATGTCCTTCCGCACGGCTGCTCTATTCGCTGGGCTGGCTCTAACTTCGTCGGCTGCTCAAGAGCAACAGCCTGTTCATGATCTAGGGTTGAACGCCTATGACCAGGCACGAGATGTGACCCTGAACGAGGTCGCG
TTCCTGACAACGACAGCATGTCATCCAAGCCTGTACAATGCGGATGTGACCAGCCGGGTCTGCTTCACGGAATTTACAACGGTGACGACGCAAACAAGTGGGGGTGGTACATACTACAAGTTTCAAGTGAAGGGCTGC
CCTGTGGACACGGAAAAACAGTTGGGCTACTGCCGCGAGGGTGCATGCTCGACGACCTCGCTCTACGAAGTCGCCATCTACTCGCAGCCGCGGACGAGCGCGGTCTTTCTGACGTCTATCAAGGGGGTAGTTTAA
```

Nucleotide alignment of *Hpa*EPIC -C:

```
       1                                                                                                       100
EMOYC  ATGATGTCCT TCCGCACGGC TGCTCTATTC GCTGGGCTGG CTCTAACTTC GTCGGCTGCT CAAGAGCAAC AGCCTGTTCA TGATCTAGGG TTGAACGCCT
EMWAC  ATGATGTCCT TCCGCACGGC TGCTCTATTC GCTGGGCTGG CTCTAACTTC GTCGGCTGCT CAAGAGCAAC AGCCTGTTCA TGATCTAGGG TTGAACGCCT
NOCOC  ATGATGTCCT TCCGCACGGC TGCTCTATTC GCTGGGCTGG CTCTAACTTC GTCGGCTGCT CAAGAGCAAC AGCCTGTTCA TGATCTAGGG TTGAACGCCT

       101                                                                                                     200
EMOYC  ACGACCAGGC ACGAGATGTG ACCCTGAACG AGGTCGCGTT CCTGACAACG ACAGCATGTC ATCCAAGCCT GTACAATGCG GATGTGACCA GCCGGGTCTG
EMWAC  ACGACCAGGC ACGAGATGTG ACCCTGAACG AGGTCGCGTT CCTGACAACG ACAGCATGTC ATCCAAGCCT GTACAATGCG GATGTGACCA GCCGGGTCTG
NOCOC  ATGACCAGGC ACGAGATGTG ACCCTGAACG AGGTCGCGTT CCTGACAACG ACAGCATGTC ATCCAAGCCT GTACAATGCG GATGTGACCA GCCGGGTCTG

       201                                                                                                     300
EMOYC  CTTCACGGAA TTTACAACGG TGACGACGCA AACAAGTGGG GGTGGTACAT ACTACAAGTT TCAAGTGAAG GGCTGCCCTG TGGACACGGA AAAACAGTTG
EMWAC  CTTCACGGAA TTTACAACGG TGACGACGCA AACAAGTGGG GGTGGTACAT ACTACAAGTT TCAAGTGAAG GGCTGCCCTG TGGACACGGA AAAACAGTTG
NOCOC  CTTCACGGAA TTTACAACGG TGACGACGCA AACAAGTGGG GGTGGTACAT ACTACAAGTT TCAAGTGAAG GGCTGCCCTG TGGACACGGA AAAACAGTTG

       301                                                                                                     400
EMOYC  GGCTACTGCC GCGAGGGTGC ATGCTCGACG ACCTCGCTCT ACGAAGTCGC CATCTACTCG CAGCCGCGGA CGAGCGCAGT CTTTCTGACG TCTATCAAGG
EMWAC  GGCTACTGCC GCGAGGGTGC ATGCTCGACG ACCTCGCTCT ACGAAGTCGC CATCTACTCG CAGCCGCGGA CGAGCGCAGT CTTTCTGACG TCTATCAAGG
NOCOC  GGCTACTGCC GCGAGGGTGC ATGCTCGACG ACCTCGCTCT ACGAAGTCGC CATCTACTCG CAGCCGCGGA CGAGCGCGGT CTTTCTGACG TCTATCAAGG

       401      411
EMOYC  AGGTAGTTTA A
EMWAC  AGGTAGTTTA A
NOCOC  GGGTAGTTTA A
```

Protein alignment of *Hpa*EPIC -C:

```
       1                                                                                                       100
EMOYC  MMSFRTAALF AGLALTSSAA QEQQPVHDLG LNAYDQARDV TLNEVAFLTT TACHPSLYNA DVTSRVCFTE FTTVTTQTSG GGTYYKFQVK GCPVDTEKQL
EMWAC  MMSFRTAALF AGLALTSSAA QEQQPVHDLG LNAYDQARDV TLNEVAFLTT TACHPSLYNA DVTSRVCFTE FTTVTTQTSG GGTYYKFQVK GCPVDTEKQL
NOCOC  MMSFRTAALF AGLALTSSAA QEQQPVHDLG LNAYDQARDV TLNEVAFLTT TACHPSLYNA DVTSRVCFTE FTTVTTQTSG GGTYYKFQVK GCPVDTEKQL

       101                              136
EMOYC  GYCREGACST TSLYEVAIYS QPRTSAVFLT SIKEVV
EMWAC  GYCREGACST TSLYEVAIYS QPRTSAVFLT SIKEVV
NOCOC  GYCREGACST TSLYEVAIYS QPRTSAVFLT SIKGVV
```

Figure S6. Sequences and alignments of *Hpa*EPIC-C from various isolates.
