# Supplementary material for: A Role in Immunity for Arabidopsis Cysteine Protease RD21, the Ortholog of the Tomato Immune Protease C14
Source: PLoS One. 2012 Jan 6;7(1):e29317. doi: 10.1371/journal.pone.0029317 (PMC3253073; doi:10.1371/journal.pone.0029317)
Supplement: Figure S7 — Both rd21-1 and rd21-2 are null mutants. Leaf extracts of Col-0 and rd21 mutant plants were labelled with DCG-04 in the presence or absence of an excess E-64 and separated on protein gels. Proteins were detected with RD21 antibody (A) and streptavidin-HRP (B), and coomassie staining (C). (PDF) [file pone.0029317.s007.pdf]

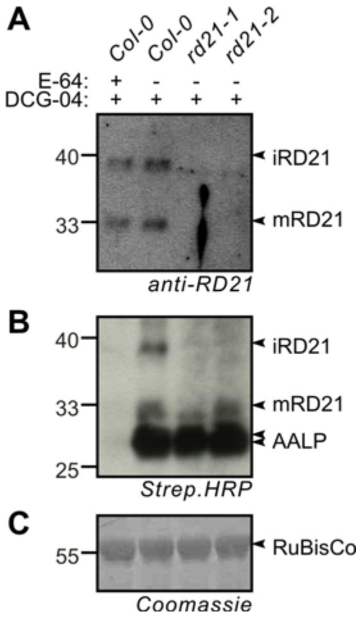

Figure S7. Both *rd21-1* and *rd21-2* are null mutants.

Leaf extracts of Col-0 and *rd21* mutant plants were labelled with DCG-04 in the presence or absence of an excess E-64 and separated on protein gels. Proteins were detected with RD21 antibody (**A**) and streptavidin-HRP (**B**), and coomassie staining (**C**).
