# Supplementary material for: A Role in Immunity for Arabidopsis Cysteine Protease RD21, the Ortholog of the Tomato Immune Protease C14
Source: PLoS One. 2012 Jan 6;7(1):e29317. doi: 10.1371/journal.pone.0029317 (PMC3253073; doi:10.1371/journal.pone.0029317)
Supplement: Figure S8 — Pinf EPICs inhibit Arabidopsis RD21. Nicotiana benthamiana leaf extracts transiently overexpressing Arabidopsis RD21 (by agroinfiltration) were preincubated (30 min) with 40 µM E-64 or with 1.2 µM PinfEPIC1, PinfEPIC2B (Tian et al., 2007) or chicken Cystatin (Sigma) and then labelled with 4 µM fluorescent DCG-04 for 2 hours. Proteins were separated on protein gels and fluorescent proteins were detected using a fluorescence scanner. (PDF) [file pone.0029317.s008.pdf]

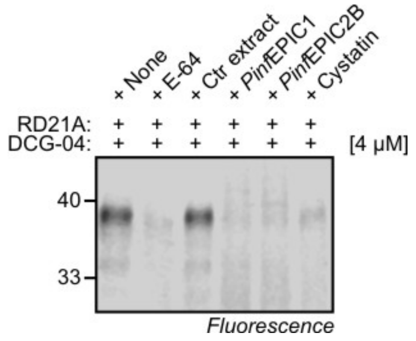

Figure S8. *Pinf*EPICs inhibit Arabidopsis RD21.

Nicotiana benthamiana leaf extracts transiently overexpressing Arabidopsis RD21 (by agroinfiltration) were preincubated (30 min) with 40 μM E-64 or with 1.2 μM PinfEPIC1, *Pinf*EPIC2B (Tian et al., 2007) or chicken Cystatin (Sigma) and then labelled with 4 μM fluorescent DCG-04 for 2 hours. Proteins were separated on protein gels and fluorescent proteins were detected using a fluorescence scanner.
