# Supplementary material for: A Role in Immunity for Arabidopsis Cysteine Protease RD21, the Ortholog of the Tomato Immune Protease C14
Source: PLoS One. 2012 Jan 6;7(1):e29317. doi: 10.1371/journal.pone.0029317 (PMC3253073; doi:10.1371/journal.pone.0029317)
Supplement: Table S1 — Sequences of primers used in this study. (PDF) [file pone.0029317.s009.pdf]

Supplemental Table S1: Sequences of primers used in this study.

**Name**	**Sequence (5'-3')**	**Direction**	**Purpose**
R203	CGAACGCCACATGTGACTCTCCCAAC	Forward	Sequencing genomic *Hpa*EPIC-A
R204	AGCGTACTCGAAGCGGCAAGGAGTGC	Reverse	
R208	CGATGGGCTGCTCCCCCCCGCCCTG	Forward	Sequencing genomic *Hpa*EPIC-B
R209	GACACGCAAAATTACCCCAACTGGC	Reverse	
R213	GCTTTCCGTATCATTGATGATGCC	Forward	Sequencing genomic *Hpa*EPIC-C
R214	GCACTTTGTGTTTGCTCTCAAAC	Reverse	
R210	GGGGAAGCTTCACCATCACCATCACCATCTACTACCAGAGCATATGCCTGACGTG	Forward	RT-PCR of *Hpa*EPIC-B
R211	GGGGCTGCAGTCATACAAGTTGCATGCTGGGG	Reverse	
R215	GGGGAAGCTTCACCATCACCATCACCATGCTCAAGAGCAACAGCCTGTTCATG	Forward	RT-PCR of *Hpa*EPIC-C
R216	GGGGCTGCAGTTAAACTACCTCCTTGATAGACGTC	Reverse	
	TATCGGATGACGATTCTTCGTGCAG	Forward	RT-PCR of *AtPP2A* (At1g13320)
	GCTTGGTCGACTATCGGAATGAGAG	Reverse	
